# The case for stronger regulation of deceptive nutrition-related claims on unhealthy food

**DOI:** 10.1371/journal.pmed.1004724

**Published:** 2025-08-28

**Authors:** Marissa G. Hall, Anna H. Grummon

**Affiliations:** 1 Department of Health Behavior, Gillings School of Global Public Health, University of North Carolina at Chapel Hill, Chapel Hill, North Carolina, United States of America; 2 Lineberger Comprehensive Cancer Center, University of North Carolina at Chapel Hill, Chapel Hill, North Carolina, United States of America; 3 Department of Pediatrics, Stanford University School of Medicine, Palo Alto, California, United States of America; 4 Department of Health Policy, Stanford University School of Medicine, Stanford, California, United States of America

## Abstract

Deceptive nutrition-related claims are pervasive on unhealthy packaged foods. This Perspective describes the potential for these claims to harm consumer health and advocates for tighter regulation of misleading claims to empower individuals to make more nutritious choices.

“Natural” vanilla ice cream. Mac and cheese topped with “real” cheddar cheese, but containing nearly a day’s worth of saturated fat. Toaster pastries boasting that they have “no high fructose corn syrup,” even though one serving contains close to the daily limit for added sugar (**Fig 1**). Spend any time in a grocery store and you will see these and many other nutrition-related claims prominently displayed on unhealthy foods. (By “nutrition-related” claims, we mean statements about the nutritional content or ingredients of a product, its health effects, or its overall healthfulness. And by “unhealthy,” we mean foods the US government defines as high in added sugar, sodium, or saturated fat). In this Perspective, we describe the harmful effects of these claims, as well as options for regulating them, using the US as a case study, while noting that similar arguments apply across the globe.

**Fig 1:**
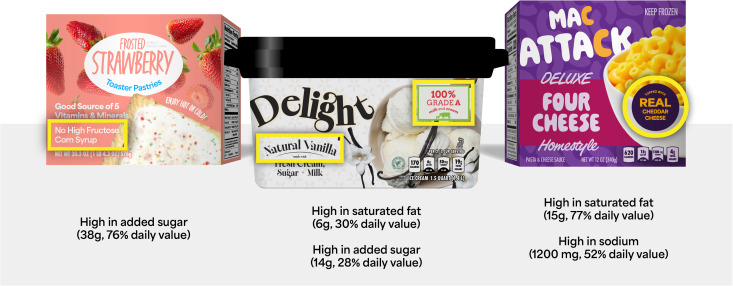
Typical examples of nutrition-related claims on foods high in added sugar, sodium, or saturated fat, modeled after real products. Claims are marked with yellow boxes. Nutritional facts information is shown per serving; 20% or more of the daily value is considered “high” by FDA.

Nutrition-related claims are ubiquitous on food. Ninety-seven percent of sugar-sweetened fruit drinks in the US display at least one nutrition-related claim such as “low calorie” or “100% vitamin C” [1]. “Natural” claims are especially common: in 2018, 27% of the breakfast cereals and 20% of the desserts, sweets, and candies purchased in the US were labeled as “natural” [2]. Currently, food companies have substantial leeway in how they use nutrition-related claims on unhealthy foods.

In theory, nutrition-related claims could help consumers make healthier food choices. But in practice, many of these claims deceive consumers or otherwise cloud their judgment about how healthy products are by generating “halo effects” in which people assume foods have positive characteristics that are unrelated to the claims (for example, thinking that “natural” ice cream has fewer calories). In our recent experiment, we showed parents of young children two identical fruit drinks: one with and one without a nutrition-related claim. These drinks are not recommended for young children because they are high in added sugar; yet nearly half of parents who viewed a fruit drink with a “100% All Natural” claim believed (incorrectly) that the drink contained no added sugar. Only 12% of parents believed so when no claim was present, a 4-fold difference [3]. Other studies have found that people incorrectly believe that products like soda and potato chips are lower in calories [4,5] and fat [5] when they display “natural” claims compared to the same products without such claims.

Regulating the use of nutrition-related claims on unhealthy foods could protect consumers from deception. The US Food and Drug Administration (FDA) is responsible for regulating most of the food supply in the US, including determining what claims are allowable, defining these claims, and enforcing compliance. The US Department of Agriculture (USDA) plays a similar role in regulating claims on meat, dairy, and eggs. The FDA and USDA can look to tobacco control efforts for a precedent on regulating deceptive claims. Until 2010, cigarette companies were allowed to advertise cigarettes and other tobacco products using claims like “light” and “mild”. But there is no such thing as a “mild” cigarette: all cigarettes are harmful to health. Recognizing that consumers were being deceived by these claims, Congress passed the Family Smoking Prevention and Tobacco Control Act, which required the FDA to prohibit these claims on tobacco products unless they meet rigorous criteria, including demonstrating that the product actually lowers health risks. So far, only a handful of tobacco products have met these criteria and been cleared to use the regulated claims. Globally, the European Union and United Kingdom require a similar process in which food companies can only use nutrition and health claims that have been approved as accurate, not misleading, and based on scientific evidence [6,7].

Similar regulations could prevent consumers from being deceived by nutrition-related claims, but the FDA and USDA need to act. One commonsense step would be to prohibit nutrition-related claims that have been shown to be deceptive from appearing on products that do not meet federally defined nutrition standards. For example, the FDA has released a formal definition of what qualifies as a “healthy” food [8] and could ban nutrition-related claims on foods that do not meet this definition. The FDA could also ban claims on foods it defines as being high in added sugar, sodium, or saturated fat (i.e., containing ≥20% of a day’s worth of these nutrients). The latter approach would mirror Mexico’s 2020 law prohibiting certain types of nutrition-related claims on products that exceed thresholds for high amounts of sugar, sodium, saturated fat, or calories [9]. The European Union has also adopted regulations that would limit the use of claims on foods not meeting nutrition standards, though these standards have not yet been set [10].

Another helpful step would be to create definitions for commonly used but potentially deceptive claims to ensure these claims can only be used in ways that promote consumer understanding. For example, “natural” claims are only loosely and informally regulated [2], meaning that they are currently allowed on many unhealthy foods. To tackle this, the FDA and USDA could create a formal definition of the term “natural” and its variations (like “naturally flavored”) and enforce the use of the terms.

In addition to preventing deception, regulating claims could also encourage consumers to choose healthier foods, ultimately leading to improved public health. A meta-analysis summarizing 17 experimental studies found that, on average, consumers have nearly two times the odds of purchasing or consuming foods that display claims compared to the same foods without them [11]. Regulating claims would allow truly healthy foods to display claims while preventing unhealthy foods from benefiting from this boost in sales. Regulating misleading claims could also lead food companies to reformulate their products to be healthier. For example, after the introduction of new “high in sugar” warning labels in Chile, the percent of products requiring a “high in sugar” label dropped from 80% to 60%, indicating that food companies changed their products to avoid having to display the “high in sugar” labels [12]. Similarly, if certain nutrition-related claims were only allowed on foods that were not high in added sugar, sodium, or saturated fat, food companies would have an incentive to lower the levels of these nutrients in their products to continue to use these claims.

Realistically, the FDA and USDA may not be able to regulate all deceptive claims, and the food industry is likely to challenge regulations. Other avenues will remain important to combat misleading claims. For example, in January 2025 the FDA proposed a new regulation that, for the first time, would require companies to display front-of-package nutrition labels indicating when their products are high in added sugar, sodium, or saturated fat [13]; about a dozen countries globally have adopted similar policies. Well-designed front-of-package nutrition labels could help counteract the deceitful influence of nutrition-related claims, and with more rigid guidelines, consumer protection advocates could bring lawsuits against the food industry for potential violations. Health communication campaigns could also educate consumers to identify and interpret deceptive claims.

Unhealthy diets cause nearly 8 million deaths globally every year [14]. Reducing diet-related disease will be a challenge. The US and other countries can start by protecting consumers from the deceptive marketing of unhealthy foods. Ice cream, mac and cheese, and toaster pastries will still be on the grocery shelves. With stronger regulation, consumers would no longer be needlessly confused about the nutritional content of these foods, but instead informed and empowered to make more nutritious choices.

## Abbreviations

**:**
FDA: Food and Drug Administration
USDA: US Department of Agriculture
